# The Effects of Perceived Stigma on the Concealment of Disease and Satisfaction with Life in Patients with Epilepsy: An Example in Eastern Turkey

**DOI:** 10.1155/2022/1064999

**Published:** 2022-02-14

**Authors:** Gülcan Bahçecioğlu Turan, Zülfünaz Özer, Beyan Özden

**Affiliations:** ^1^Department of Nursing, Faculty of Health Sciences, Fırat University, Elazığ, Turkey; ^2^Department of Nursing, Faculty of Health Sciences, Istanbul Sabahattin Zaim University, Istanbul, Turkey; ^3^Firat University Health Sciences Institute, Nursing Department, Elazığ, Turkey

## Abstract

**Background:**

Stigma and exclusion are common features of epilepsy in both developed and developing countries, and they cause a significant burden associated with the condition. At the same time, although it varies from country to country depending on cultural differences and economic conditions, having epilepsy causes significant social consequences.

**Objective:**

This study was conducted to examine the effects of perceived stigma on the concealment of disease and satisfaction with life in patients with epilepsy living in the east of Turkey.

**Methods:**

This cross-sectional and descriptive study was carried out with 150 patients who met the study criteria and who agreed to participate in the study between March and July 2021 in a university hospital in Elazığ, east of Turkey. The data were collected using a personal information form, an Epilepsy Stigma Scale (ESS), a Concealment of Epilepsy Scale (CES), and a Satisfaction with Life Scale (SWLS).

**Results:**

The total mean ESS score of the patients was 40.7 ± 9.04, the total CES mean score was 57.19 ± 12.57, and the total SWLS mean score was 6.68 ± 2.86. When the regression coefficients were examined, it was found that the ESS variable had a positive and significant effect on the CES, while the ESS and the CES had a negative and significant effect on satisfaction with life (*p* < 0.001).

**Conclusion:**

It was found that the patients had high levels of perceived stigma and concealment of epilepsy and low satisfaction with life levels. It was also found that the patients concealed their disease for fear of stigma, which negatively affected their satisfaction with life.

## 1. Introduction

Epilepsy is the most common noncontagious chronic brain disease, and it affects individuals of all ages. It is estimated that more than 50 million people in the world and approximately 750 thousand people in Turkey have epilepsy. Approximately 80% of these patients live in low- and moderate-income level countries [1, 2]. It is a known fact that when diagnosed and treated correctly, the disease can be controlled in 70% of individuals living with epilepsy, and these individuals may lead a seizure-free life. However, patients with epilepsy throughout the world still have to fight with “stigmatization” brought by prejudice and discrimination [1, 3].

Stigma discredits individuals because others consider these individuals to be inferior and thus disparage them [4]. According to Goffman, stigma is “a deeply discrediting attribute,” and discredited individuals are typically socially rejected [5]. Scambler and Hopkins defined two types of stigma related to epilepsy: enacted stigma and felt stigma. Enacted stigma refers to real cases of discrimination due to epilepsy, while felt stigma refers to the shame of having epilepsy and the fear of facing the enacted stigma associated with it [6]. Common views defining epilepsy as a supernatural mental disease or a punishment of sins have led to an increase in felt stigma in developed or developing countries. Increased stigma disrupts individuals' self-management of epilepsy and causes social isolation and the concealment of the disease in patients with epilepsy [3, 7]. The concealment of the disease is the first reflection of epilepsy-related stigma. With the fear of negative evaluations and stigma, patients attempt to conceal their disease from nonfamily members [8, 9]. Sometimes, patients may even conceal the disease from family members [10] and their future spouses due to the negative effects of discrimination on marriage [11, 12]. In some situations, the feeling of shame caused by internalized stigma may cause individuals with stigma to conceal their disease [13]. In a large number of studies conducted in Turkey, it has been found that individuals with epilepsy are frequently faced with social and cultural stigmatization. It has also been found in these studies that individuals conceal their diseases due to social stigmatization [3, 7–9]. Many globally conducted studies have indicated that the health and well-being of individuals stigmatized due to epilepsy are negatively affected. These individuals frequently experience fear, desperation, anxiety, depression, low self-respect, low quality of life, and low satisfaction with life [14–16]. Satisfaction with life is the state of well-being in different aspects of one's life, which encompasses satisfaction, happiness, and spirit [17]. It is determined by the holistic evaluation of one's physical, psychological, social, and economic quality of life and the type and number of seizures [18].

Stigma and exclusion are common features of epilepsy in both developed and developing countries, and they cause a significant burden associated with the condition. At the same time, although it varies from country to country depending on cultural differences and economic conditions, having epilepsy causes significant social consequences [19]. When all these are considered, although a limited number of studies have been conducted on perceived stigmatization in patients with epilepsy in some regions of Turkey [3, 7, 8, 17], no studies have been found which examined the effects of perceived stigmatization in patients with epilepsy living in the east of Turkey, known to have low socioeconomic level, on concealing the disease and life satisfaction. For this reason, this study aims to fill in a gap in the literature by examining the effects of perceived stigmatization on concealing the disease and life satisfaction in patients with epilepsy living in the east of Turkey.

### 1.1. Research Questions

- What is the perceived level of stigma in patients with epilepsy?
- What is the level of concealment of the disease in patients with epilepsy?
- What is the level of life satisfaction in patients with epilepsy?
- Does perceived stigma affect disease concealment and satisfaction with life in patients with epilepsy?

## 2. Materials and Methods

### 2.1. Type of Study

This study has a cross-sectional and descriptive design.

### 2.2. Time and Place of Study

The study was conducted in the neurology outpatient clinic of a university hospital in Elazığ, a city in eastern Turkey, between March and July 2021.

### 2.3. Sample and Population of Study

The population of the study consisted of patients with epilepsy who referred to the neurology outpatient clinic of Fırat University Hospital in Elazığ, which is in the east of Turkey, between March and July 2021. The sample of the study was calculated based on the study by Dayapoğlu et al. [14] (*t*-test for age-independent groups). As a result of the evaluations, the sample was calculated as *d* = 0.619 (moderate effect size), power = 0.95, and *α* = 0.05 in G^*∗*^Power 3.1.9.2 statistical program. According to this, the minimum number of patients to be included in the study was determined as 116. 187 patients were reached within these dates. The study was completed with 150 patients since 30 of these patients did not meet the study criteria and 7 of the patients did not want to participate in the study.

#### 2.3.1. Inclusion Criteria

- Being older than 18 years of age
- Ability to communicate adequately
- Not having a psychiatric problem
- Having a definitive diagnosis of epilepsy
- Having been diagnosed with epilepsy at least for 6 months

### 2.4. Data Collection Instruments

The data were collected by the researcher using the face-to-face interview technique (required precautions were taken considering the pandemic process) using the “Descriptive Information Form,” “Epilepsy Stigma Scale,” “Concealment of Epilepsy Scale,” and “Satisfaction with Life Scale.” The total time for data filling is 10–15 minutes.

#### 2.4.1. Personal Information Form

This form prepared by the researchers includes 14 questions as patients' age, gender, marital status, level of education, employment status, income status, family structure, diagnosis year, the state of having seizures during the past year, the state of having another chronic disease, seizure type, the state of using an antiepileptic drug, the number of drugs used, and the state of concealing the epilepsy disease.

#### 2.4.2. Epilepsy Stigma Scale (ESS)

The Epilepsy Stigma Scale, which Aydemir et al. [7] developed to evaluate perceived stigmatization in patients with epilepsy, was used in the study. The scale has 10 questions, including five Likert questions. The items in the scale are scored from one for “totally disagree” to five for “totally agree.” The minimum possible score from the scale is 10, while the maximum possible score is 50. High scores show a high feeling of stigmatization. Cronbach's alpha value for the scale was reported as 0.86 [7]. In this study, Cronbach's alpha value was 0.92.

#### 2.4.3. Concealment of Epilepsy Scale (CES)

The Concealment of Epilepsy Scale, which Aydemir et al. [7] developed, was used to evaluate the concealment of disease in patients with epilepsy who participated in the study. The scale has 17 questions, including five Likert questions. The items in the scale are scored from one for “totally disagree” to five for “totally agree.” The minimum possible score from the scale is 17, while the maximum possible score is 85. High scores show a higher potential to conceal epilepsy. Cronbach's alpha value of the scale was reported as 0.92 [7]. In our study, Cronbach's alpha value was 0.87.

#### 2.4.4. Satisfaction with Life Scale (SWLS)

The Satisfaction with Life Scale is a five-item, seven-point Likert-type scale developed to measure life satisfaction [20]. Yetim conducted a Turkish validity and reliability study of the scale [21]. The minimum possible score from the scale is 5, while the maximum possible score is 35. A higher score on the scale indicates greater satisfaction with life. Cronbach's alpha value of the scale was reported as 0.86 [21]. In our study, Cronbach's alpha value was 0.85.

### 2.5. Evaluation of Data

The data were evaluated with SPSS 22 programs. Descriptive statistics concerning the variables were shown as percentage, number, standard deviation, and mean. A normality test of the data was performed since the skewness and kurtosis values were not between −2 and +2, and it was found that the data did not show a normal distribution [22]. Regression analysis was used in the data analysis. A *p* < 0.05 value was considered statistically significant in all statistical comparisons.

### 2.6. Ethical Considerations

Ethical approval from the Non-Interventional Ethics Committee of a university (2021/02–29 numbered, 04.02.2021 dated) and permission from the institution in which the study was conducted were obtained for this study. The form, which included the required information about the aim and application method of the study, was sent online to individuals participating in the study, and their approval was obtained. This study was carried out in line with the ethical standards of the Declaration of Helsinki. Participants who volunteered were included in the study, and personal identity information was kept confidential.

## 3. Results

The mean age of the patients was 31.74 ± 10.39 years, and the mean duration of disease was 6.82 ± 6.01. Of the patients, 56.7 were male, 40.7% were married, 40.7% were primary education graduates, 65.3% were not working, 60% had incomes lower than their expenses (low level of income), and 89.3% had a nuclear family. It was found that 40.7% of the patients had experienced one seizure in the past year, 40% had experienced a focal onset seizure, 64% did not have another chronic disease, 100% used antiepileptic drugs, 76% used more than one drug, and 57.3% concealed their epilepsy (Table 1).

It was found that the mean total ESS score of the patients was 40.7 ± 9.04, the mean total CES score was 57.19 ± 12.57, and the mean total SWLS score was 6.68 ± 2.86 (Table 2).

Simple linear regression analysis conducted to find out the effect of Epilepsy Stigma Scale and CES variables on SWLS was found to be statistically significant (*F* = 9.933, *p* < 0.001). The variables in the model explain 29.4% of the total variance in satisfaction with life (*p* < 0.001). When the regression coefficients were examined, it was found that ESS (*β* = −0.559, *p* < 0.001) and CES (*β* = −0.428, *p* < 0.001) had a negative and significant effect on satisfaction with life (Table 3). Simple linear regression analysis conducted to find out the effect of ESS on CES was found to be statistically significant (*F* = 88.554, *p* < 0.001). ESS in the model explains 37% of the total variance of CES (*p* < 0.001). When the regression coefficients are examined, it can be seen that ESS (*β* = 0.612. *p* < 0.001) variable has a positive and significant effect on CES (Table 3).

## 4. Discussion

This study was conducted to examine the effects of felt stigma in patients with epilepsy on the concealment of the disease and satisfaction with life. The results obtained were discussed in light of the literature. Mean total ESS scores in the study were found to be high. The felt stigma levels of the patients were found to be high. A study that Aydemir et al. [8] conducted on 200 patients with epilepsy in Turkey found that these patients had high stigma perceptions. In another study, Tedrus et al. [23] reported that patients with epilepsy had high perceived stigma levels. Different studies have also indicated that patients with epilepsy have high perceived stigma levels [20, 24–27]. This study's results were similar to those in the literature. When an individual has a disability, disorder, or disease, the stigma cycle can begin. Feeling a lack of confidence and skill can become inevitable in an individual who experiences isolation in social life by feeling stigmatized. All of these may lead to the restriction of activity and social roles in individuals. Patients with epilepsy live without knowing when and where their seizures will occur, whether they will lose consciousness, and whether their seizures can be controlled. This uncertainty makes individuals sensitive, fragile, and vulnerable to social situations and, in turn, causes them to have higher perceived stigma [28]. It has been reported that stigma in patients with epilepsy causes as much harm as the disease itself [10].

This study found that the patients had moderate mean total CES scores. It also found that the patients had a moderate tendency to conceal the disease. In a study conducted in eastern Turkey, Dayapoglu et al. [14] determined that patients with epilepsy had a moderate tendency to conceal the disease. Another study conducted in Turkey found that patients with epilepsy have a moderate tendency to conceal the disease [11]. This study's results were similar to those in the literature. An epilepsy diagnosis may have negative consequences for patients, for their families, and indirectly for society. Individuals with epilepsy may have difficulty adapting to social norms, confronting society, finding a job, and starting a family. This situation causes individuals to conceal the disease [23]. In addition, the present study found that as the patients' level of felt stigmatization increased, their tendency to conceal the disease also did. In a study conducted, it was reported that almost half of the Turkish patients with epilepsy concealed their diseases and most did this in their diagnosis and this was a long-term strategy to cope with stigmatization. Also, in the same study, almost 90% of the patients stated that the most important reason why they concealed the disease was feeling stigma [29]. Various studies conducted in Turkey have indicated that individuals with epilepsy frequently face cultural and social stigma, and social stigmatization leads patients to conceal their disease [7, 9]. In different studies conducted, it has been accepted that concealment of the disease is a part of the stigma. It has been stated that the concealment of the disease is associated with stigma and, in fact, predicts stigma [3, 8, 30]. Patients with epilepsy often face social stigma, and this is one of the most important factors causing patients to conceal their disease [10]. Even if individuals with epilepsy have not faced social stigma, their internalized stigma may cause them shame, and they may conceal their disease [13]. Therefore, most patients do not disclose their disease, few are generally informed about their condition and adopt disease concealment as the first strategy to fight enforce stigma [7].

In this study, the mean SWLS total scores of the patients were low. It was found that the patients had very low satisfaction with life. It was also found that a postgraduate degree and good economic status had a positive effect on satisfaction with life, while having a history of five or more seizures in the past year and having another chronic disease negatively impacted it. No studies that examined satisfaction with life among patients with epilepsy were found in the literature. Since satisfaction with life is a process in which quality of life is evaluated according to the criteria an individual prefers [31], the results of the study were evaluated with studies conducted on quality of life. A study by Tedrus et al. [27] indicated that patients with epilepsy have a low quality of life. A systematic review showed that individuals with epilepsy have a low quality of life and that a low level of education and socioeconomic status and the frequency and intensity of seizures have a further negative effect on it [32]. It has also been reported that patients with epilepsy in Turkey have a low quality of life [18]. The unpredictable nature of epilepsy is a factor that negatively affects harmony about life satisfaction. While all chronic diseases affect individuals' quality of life, the impact may be more evident in individuals with epilepsy because they must limit specific aspects of their lives based on the intensity of their epileptic seizures [28]. Such limitations may impact patients' satisfaction with life.

This study found that felt stigma and concealment of the disease negatively impacts satisfaction with life. Studies have shown that felt stigma and the concealment of the disease negatively affect patients' lives (e.g., their future profession, social life, marriage, and decision to have children) [8, 33]. Another study found that felt stigma negatively affects social well-being and quality of life [24]. In a study conducted with patients with epilepsy, Tedrus et al. [27] concluded that felt stigma has a negative effect on quality of life. Felt stigma significantly affects quality of life, especially in low- and moderate-income societies [34]. In a study conducted with 140 patients, 70 of whom were patients with resistant epilepsy, Viteva [35] reported a negative association between high stigma rates and quality of life. The higher the felt stigma rate, the lower the quality of life. A different study found that felt stigma is the most important predictor of poor quality of life [25]. In a study considering the psychosocial aspect of epilepsy, Yeni et al. [3] showed that felt stigma had a significant effect on life quality. A meta-analysis showed that the most important factor impacting quality of life in patients with epilepsy was stigma [26]. A systematic review of regression studies found that felt stigma is the most important predictor of poor quality of life [20]. The results of this study were similar to those of the literature. Social stigma, which significantly affects quality of life, has been mentioned alongside epilepsy throughout history. Being stigmatized as an epilepsy patient is considered worse than having a seizure; therefore, patients and families have concealed the disease for centuries [18]. It is thought that stigmatization causes social isolation in patients and contributes to low satisfaction with life in patients.

### 4.1. Limitations of the Study

The first limitation of our study is that the results of the study are only valid for patients included in the study; therefore, they cannot be generalized. The second limitation is that the fear of being negatively evaluated by society can differ significantly among individuals with different religious and cultural traditions. Thus, care should be taken when interpreting the results in different religious and cultural environments. The reliability of data is also limited by the accuracy of the answers that the study participants provided. The third limitation, reliability of data, is associated with the accuracy of the responses given by all the patients in the study. The fourth limitation is the fact that the study was conducted in a single public hospital because there are two public hospitals in the city where the study was conducted. Only one public hospital allowed for the study to be conducted. The fifth limitation is the fact that the data were filled in face-to-face by the researcher.

## 5. Conclusion

This study found that patients had high felt stigmatization levels, moderate concealment of the disease levels, and low satisfaction with life levels. It showed that patients concealed their disease with the fear of stigmatization, and this situation had a negative effect on their satisfaction with life. Individuals with epilepsy having negative thoughts about themselves should be addressed. Practical solutions should be developed for stigmatization and the concealment of the disease that increase satisfaction with life. It is recommended that in addition to treating individuals with epilepsy, health professionals should evaluate felt stigma, concealment of disease, and satisfaction with life in patients within specific intervals and should conduct the study with patient groups in different regions.

## Figures and Tables

**Table 1:** Sociodemographic and disease characteristics of the patients.

		Mean ± SD	Min–max (median)
Age		31.95 ± 10.18	18–69 (29)
Duration of disease		6.82 ± 6.01	1–28 (5)
		*n*	%

Gender	Female	65	43.3
	Male	85	56.7

Marital status	Married	61	40.7
	Single	89	59.3

Level of education	Literate	20	13.3
	Primary education	61	40.7
	High school	46	30.7
	Postgraduate	23	15.3

Working status	Working	52	34.7
	Not working	98	65.3

Level of income	Income < expense (low level of income)	90	60.0
	Income = expense (moderate level of income)	47	31.3
	Income > expense (high level of income)	13	8.7

Family type	Nuclear	134	89.3
	Extended	16	10.7

Seizures within the past year	None	14	9.3
	Once	61	40.7
	Twice	57	38.0
	5 times and more	18	12.0

Type of seizure	Focal onset	60	40.0
	Unknown onset	32	21.3
	Generalized onset	58	38.7

Presence of another chronic disease	Yes	54	36.0
	No	96	64.0
Antiepileptic drug use	Yes	150	100.0

Number of drugs used	Single	36	24.0
	Multiple	114	76.0

State of concealing epilepsy	Yes	86	57.3
	No	64	42.7

**Table 2:** Mean ESS^*∗*^, CES^*∗∗*^, and SWLS^*∗∗∗*^ measurements of the patients.

	Mean ± SD	Min–max
Epilepsy Stigma Scale	40.7 ± 9.04	12–50
Concealment of Epilepsy Scale	57.19 ± 12.57	22–76
Satisfaction with Life Scale	6.68 ± 2.86	4–18

^*∗*^ESS: Epilepsy Stigma Scale; ^*∗∗*^CES: Concealment of Epilepsy Scale; ^*∗∗∗*^SWLS: Satisfaction with Life Scale.

**Table 3:** The results of regression analysis.

Model	Variables	Univariable				
		*B*	S. error	Standard (B)	*t*	*p* value
SWLS	ESS^*∗∗∗∗*^	−0.177	0.022	−0.559	−8.198	0.001^*∗∗∗*^
	CES	−0.98	0.017	−0.428	−5.766	0.001^*∗∗∗*^
CES	ESS	0.850	0.090	0.612	9.410	0.001^*∗∗∗*^
^**∗∗∗**^p<0.01						

^*∗*^CES: Concealment of Epilepsy Scale; ^*∗∗*^SWLS: Satisfaction with Life Scale; ^*∗∗∗∗*^ESS: Epilepsy Stigma Scale.

## Data Availability

The data that support the findings of this study are available on request from the corresponding author. The data are not publicly available due to privacy or ethical restrictions.

## Additional Points

*What Is Known.* It is a known fact that when diagnosed and treated correctly, the disease can be controlled in 70% of individuals living with epilepsy and these individuals may lead a seizure-free life. However, patients with epilepsy throughout the world still have to fight with “stigmatization” brought by prejudice and discrimination. *What Is New.* It is the first study to examine the effect of perceived stigma on hiding the disease and life satisfaction in epilepsy patients living in eastern Turkey, which is known to have a lower socioeconomic level compared to other regions. It showed that patients concealed their disease with the fear of stigmatization, and this situation had a negative effect on their satisfaction with life.
